# The impact of cognitive aids on resuscitation performance in in-hospital cardiac arrest scenarios: a systematic review and meta-analysis

**DOI:** 10.1007/s11739-022-03041-6

**Published:** 2022-08-29

**Authors:** Francesco Corazza, Elena Fiorese, Marta Arpone, Giacomo Tardini, Anna Chiara Frigo, Adam Cheng, Liviana Da Dalt, Silvia Bressan

**Affiliations:** 1 Division of Pediatric Emergency Medicine, University Hospital of Padova, Padova, Italy; 2 Department of Women’s and Children’s Health, University of Padova, Padova, Italy; 3 Biostatistics, Epidemiology and Public Health Unit, Department of Cardiac, Thoracic, Vascular Sciences and Public Health, University of Padova, Padova, Italy; 4 Departments of Paediatrics and Emergency Medicine, Alberta Children’s Hospital, University of Calgary, Calgary, Canada

**Keywords:** Heart arrest, Cognitive aid, Support tool, Resuscitation, Simulation training

## Abstract

Different cognitive aids have been recently developed to support the management of cardiac arrest, however, their effectiveness remains barely investigated. We aimed to assess whether clinicians using any cognitive aids compared to no or alternative cognitive aids for in-hospital cardiac arrest (IHCA) scenarios achieve improved resuscitation performance. PubMed, EMBASE, the Cochrane Library, CINAHL and ClinicalTrials.gov were systematically searched to identify studies comparing the management of adult/paediatric IHCA simulated scenarios by health professionals using different or no cognitive aids. Our primary outcomes were adherence to guideline recommendations (overall team performance) and time to critical resuscitation actions. Random-effects model meta-analyses were performed. Of the 4.830 screened studies, 16 (14 adult, 2 paediatric) met inclusion criteria. Meta-analyses of eight eligible adult studies indicated that the use of electronic/paper-based cognitive aids, in comparison with no aid, was significantly associated with better overall resuscitation performance [standard mean difference (SMD) 1.16; 95% confidence interval (CI) 0.64; 1.69; *I*^2^ = 79%]. Meta-analyses of the two paediatric studies, showed non-significant improvement of critical actions for resuscitation (adherence to guideline recommended sequence of actions, time to defibrillation, rate of errors in defibrillation, time to start chest compressions), except for significant shorter time to amiodarone administration (SMD − 0.78; 95% CI − 1.39; − 0.18; *I*^2^ = 0). To conclude, the use of cognitive aids appears to have benefits in improving the management of simulated adult IHCA scenarios, with potential positive impact on clinical practice. Further paediatric studies are necessary to better assess the impact of cognitive aids on the management of IHCA scenarios.

**Supplementary Information:**

The online version contains supplementary material available at 10.1007/s11739-022-03041-6.

## Introduction

Despite improvements in cardiac arrest (CA) outcomes over the last couple of decades, clinical management remains challenging with low survival to hospital discharge for both out-of-hospital CA (OHCA) and in-hospital CA (IHCA). Survival rates are lower in adults compared with children (4.5–10% [1, 2] for OHCA and 20–25% [3] in IHCA compared with 4%-17.7% [1, 4, 5] and 27%–43% respectively) [6–8]. To optimize the management of CA and improve patients’ clinical outcomes, international resuscitation organisations periodically update and publish evidence-based resuscitation guidelines [6, 9–11]. Lay rescuers and healthcare providers, however, often struggle to adhere to resuscitation guidelines during management of CA [12–22], negatively impacting patient prognosis and outcomes [23].

Several educational strategies and novel technologies have been designed to assist healthcare providers in improving their adherence to guidelines for the management of CA [24–29]. Recently, different cognitive aids, in the format of paper-based and digital resources, have been developed for both lay rescuers and healthcare providers to support the management of OHCA and IHCA [30–40]. Systematic reviews on audio/video guidance and smartphone applications (apps) developed to support bystanders in managing OHCA showed that the use of these tools was associated with improved quality of bystanders’ cardiopulmonary resuscitation (CPR) [39–41]. However, the effectiveness of cognitive aids designed to assist health professionals in the medical management of IHCA remains uncertain.

The preliminary results of a recent systematic review conducted by the International Liaison Committee on Resuscitation (ILCOR) [42] showed that there are no studies evaluating the impact of cognitive aids in the management of CA in the real-life environment. In this study, we aimed to assess the effectiveness of paper-based (e.g. pocket cards, posters, or checklists) or electronic cognitive aids (e.g. applications/software for smartphones, tablets, laptops or augmented reality glasses) in improving the management of IHCA in the simulation setting. The use of cognitive aids during these uncommon and complex emergencies is challenging to assess in the natural environment and therefore simulation studies allow the generation of knowledge that can be transferred to clinical practice.

## Methods

This systematic review and meta-analysis was performed following the Preferred Reporting Items for Systematic Reviews and Meta-Analyses (PRISMA) guidelines [43]. The study protocol was registered in PROSPERO (CRD42020207323).

### Search strategy

The bibliographic search focussed on three main concepts: cardiac arrest, cognitive aid, and simulation. The search strategy was composed of subject headings and keyword terms, translated and adjusted to the syntax of each of the searched databases: MEDLINE (PubMed Interface), Embase (OVID interface), the Cochrane Library and CINAHL. In addition, the United States clinical trials registry and the Cochrane Central Register of Controlled Trials were also searched for unpublished completed trial reports, using the same search terms. The World Health Organization International Clinical Trials Registry Platform (ICTRP) could not be explored due to heavy traffic generated by the COVID-19 outbreak. The databases searches were limited to publications from 1974, when the first guidelines on resuscitation were published [44], until December 31, 2021. The detailed comprehensive search strategies and the message indicated by the ICTRP portal are reported in the supplementary material (Supplementary files 1 and 2, respectively).

### Study selection

We selected randomized controlled trials, non-randomized controlled trials, and observational studies with a comparative group, which involved healthcare professionals (physicians, residents, medical students, nurses) managing simulated scenarios of adult or paediatric IHCA with or without the use of a cognitive aid or comparing different types of cognitive aids.

The intervention of interest was the use of a cognitive aid, defined as the “presentation of prompts aimed to encourage recall of information to increase the likelihood of desired behaviours, decisions, and outcomes” [45]. Cognitive aids could be of any format, comprising paper-based types, such as pocket cards, pamphlets, checklists, posters, and laminated cards, and electronic mobile applications/software made available on different devices, such as mobile phones, smartphones, tablets, laptops, computers, and augmented reality glasses.

We included studies where the comparison(s) to the intervention were either: (i) no use of cognitive aids or (ii) the use of an alternative cognitive aid.

We excluded: (i) animal studies; (ii) incomplete/ongoing or unpublished studies (trial protocols, abstract/posters published only in conference/congress proceedings); (iii) studies assessing cognitive tools that support a single resuscitation task [e.g. feedback devices to guide depth/rate of chest compressions (CC); electronic support to exclusively guide drug preparation/administration]; (iv) studies assessing cognitive aids exclusively used as training/educational tools; (v) studies about neonatal resuscitation and (vi) studies exclusively about telemedicine, not assessing cognitive tools. No language or other restrictions were applied. Studies in languages other than English or Italian, if judged relevant after reading the abstract, were translated and evaluated.

Our primary outcomes were (i) overall team performance and adherence to guideline recommendations measured by novel or validated scoring systems/tools/checklists assessing team performance in managing the CA scenario or by pre-defined errors in actions (such as drug administration or defibrillations), delays or lack of performance of critical actions for resuscitation; and (ii) time to perform critical actions for resuscitation (i.e. initial clinical check for CA, start of CPR, start of ventilation, drug administration, defibrillation). The secondary outcomes of interest were: (i) CPR quality metrics (CC mean rate, mean depth, mean recoil, and CC/no-flow fraction or percentage of CPR compliance to guideline recommendations, no-blow fraction/ventilation fraction); (ii) non-technical skills; (iii) evaluation of cognitive aid usability; and (iv) evaluation of users’ perceived workload.

Relevant studies were identified through a three-stage process. First, two independent reviewers (EF and MA) performed a screening based on publication titles and abstracts. Then, to determine final inclusion, two additional independent reviewers (FC and GT) reviewed the full texts of the studies identified through the screening. Any disagreement at both stages was resolved by a third independent reviewer (SB). We used the Covidence systematic review management software [46] for these selection processes. We also hand-searched the reference lists of finally included articles to ensure key articles had not been overlooked.

### Data collection process

Two authors (FC and EF) independently extracted information from the finally included articles in a standard report form prepared ad-hoc for this study. The following information was extracted: study design, characteristics of the study population, type of scenario (paediatric or adult cardiac arrest; shockable/non-shockable rhythm), details on the intervention and control, and outcome measures. Study authors were contacted to obtain relevant missing data. Results were presented for available data only.

### Quality assessment and risk of bias in individual studies

Two authors (FC and GT) independently assessed each included study using the ‘Cochrane Risk of Bias’ RoB 2.0 tool [47]. Any difference in opinion between the two authors were settled by a third reviewer (SB). Due to the nature of the intervention, “blinding of participants and personnel” and “blinding of outcome assessors” was not possible. Consequently, the “blinding of participants and personnel” domain was excluded from the quality assessment and the “blinding of outcome assessors" domain was considered as the "blinding of statisticians” domain. In the domain “other sources of bias” we assessed the presence of a published/available protocol, its level of detail and the concordance between the protocol and the methods/results of the study.

### Statistical analysis

When ≥ 2 adult studies adopting the same type of intervention and comparators reported at least a measure of central tendency and a measure of dispersion for results on any of the outcomes of interest we performed a meta-analysis. Meta-analyses of adult studies were stratified by the type of cognitive aids assessed. Similarly, results of paediatric studies were combined in a meta-analysis when they had sufficient similarities in study design, participants, type of interventions and comparators, and outcome measures.

We planned to summarize dichotomous outcomes, as risk ratios (RR) with 95% CI. Standardized mean differences (SMD) and 95% CI were used for studies reporting continuous outcomes. Meta-analyses were performed using the random effects model due to the high heterogeneity of the studies. We used the *I*^2^ statistics to assess the heterogeneity between studies. Due to the limited number of studies for which meta-analyses could be performed, only one subgroup analysis by type of cognitive aid was undertaken. All analyses were conducted using *R* (version 4.0.2.).

## Results

**Fig. 1:**
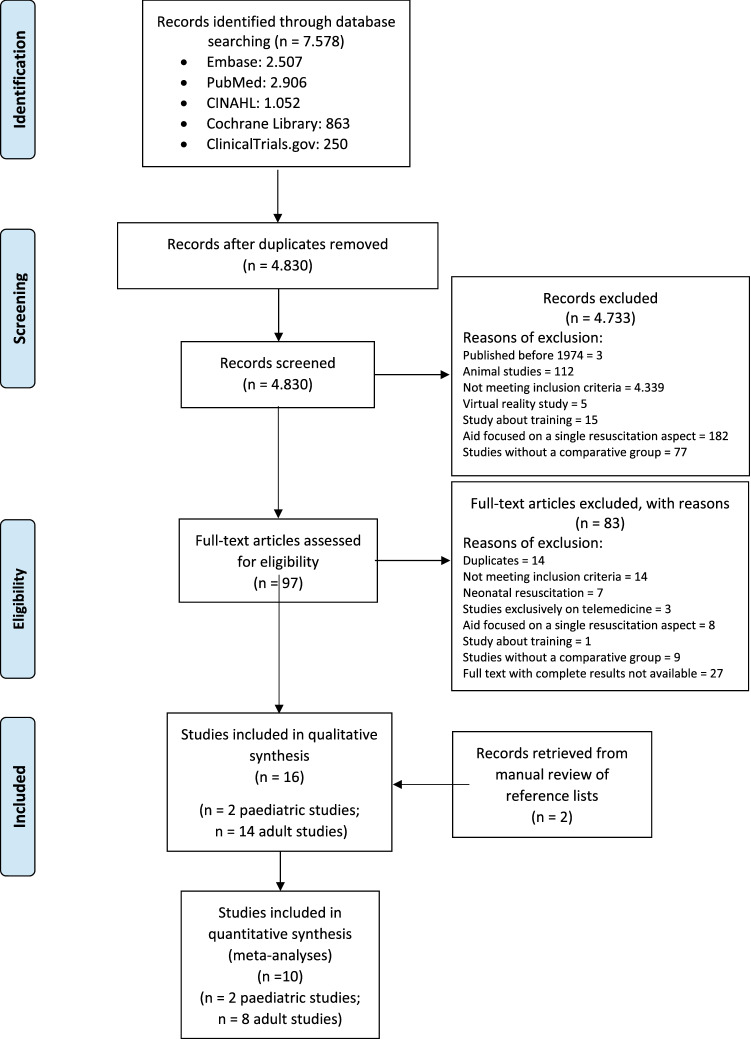
PRISMA Flow Diagram of study selection

Of the 4224 screened studies, 16 met our inclusion criteria: 14 adult CA studies [32–36, 48–56] (for a total of 688 scenarios) and two paediatric CA studies [30, 31] (for a total of 46 scenarios). The PRISMA flow chart for the study selection process is shown in Fig. 1.

## Adult studies

### Studies characteristics

The characteristics of the 14 included adult studies are shown in Table 1.

**Table 1:** Characteristics of included studies

First Author; Year	Country	Study design	Participants	Intervention	Control	Number of scenarios per group (Intervention/control)	Case scenario
Adult							
Schneider AJ; 1995^50^	USA	RCT (2 arms)	39 anaesthesia residents	‘Helper' (computer-based prompting device)	No device	20/19	VF, II degree AVB
Low D; 2011^32^	UK	RCT (2 arms)	31 ALS-trained paediatric/ emergency/anaesthesia/surgery residents	Smartphone app (iResus)	No cognitive aid	16/15	PEA → VF
Arriaga AF; 2013^35^	USA	RCT (crossover)	67 participants: anaesthesia/surgery attending physicians and residents, nurses, surgical technologist	Paper-based crisis checklist provided in booklet form	No crisis checklist	32/36	asystole, VF, unstable arrythmia
Field LC; 2014^36^	USA	RCT (crossover)	47 ACLS certified senior medical students	Electronic DST installed on iPod Touch	No DST	47/47	Unstable bradycardia, VF, asystole, pVT, PEA
Lelaidier R; 2017^51^	France	RCT (crossover)	46 anaesthesia residents	Smartphone application MAX (Medical Assistance eXpert)	No MAX app	8/8	VF
Donzé P; 2019^33^	Canada	RCT (3 arms)	57 anaesthesia residents and consultants	Smartphone application MAX (Medical Assistance eXpert)	A) paper cognitive aid B) no cognitive aid	20/19(A), 18(B)	VF
Jones I; 2019^52^	UK	NR-CT (crossover)	34 participants: student nurses, BLS-certified nurses, ALS-certified nurses	Electronic decision support system (eDSS): software, in a handheld tablet device	No eDSS	8/8	VF, PEA
Shear TD; 2019^53^	USA	RCT (2 arms)	34 anaesthesia residents	Dynamic electronic cognitive aid with embedded clinical decision support (dCA)	Static cognitive aid (laminated cards)	15/19	VF
Crabb DB; 2020^54^	USA	RCT (crossover)	56 participants: EM attending physicians/residents, nurses, critical care technicians and paramedics	ACLS CDDS: an interactive, web-based application on a large screen	No CDDS	16/16	VF, pVT, asystole, PEA
Hejjaji V; 2020^55^	USA	RCT (crossover)	53 internal medicine residents	Smartphone application “Redivus Code Blue”	No Redivus Code Blue app	53/53	pVT, VF, PEA
Hall C; 2020^56^	Australia	RCT (crossover)	75 participants: doctors and nurses	Emergency Protocols Handbook: contains 15 adult and 12 paediatric pathways	No Emergency Protocols Handbook	11/10	pVT
Koers L; 2020^34^	Netherlands	RCT (2 arms)	144 participants: general surgeons, gynaecologists, urologists, nurses	CAMDS (paper-based cognitive aids for the management of deteriorating surgical patients)	No CAMDS	25/25	2 different scenarios: shockable and non-shockable CA
Grundgeiger T;2021^48^	Germany	RCT (2 arms)	134 participants: emergency physicians and nurses	CA tablet app	No CA app	32/31	VF
Urman RD;2021^49^	USA	RCT (crossover)	304 anesthesiologists	Emergency Manual (modified version of Stanford Emergency Manual)	No Emergency Manual	29/32	2 different scenarios of PEA
Paediatric							
Siebert JN; 2017^30^	Switzerland	RCT (2 arms)	20 paediatric residents	Augmented reality glasses (Google glass) with PALS guidelines	PALS pocket reference card	10/10	pVT
Siebert JN; 2020^31^	Switzerland	RCT (2 arms)	26 paediatric residents	Tablet app “Guiding Pad”	PALS pocket reference card	13/13	pVT

*ACLS* advanced cardiac life support, *ALS* advanced life support, *AVB* atrioventricular block, *CA* cardiac arrest, *CDDS* Clinical Decision Display System, *DST* decision support tool, *EM* Emergency Medicine, *NR-CT* non-randomized control trial, *PALS* paediatric advanced life support, *PEA* = pulseless electrical activity, *pVT* pulseless ventricular tachycardia, *RCT* = randomized controlled trial, *UK* United Kingdom, *USA* United States of America, *VF* ventricular fibrillation

The articles were published between 1995 and 2021, with nine (64%) published in the past three years [33, 34, 48, 49, 52–56]. Thirteen studies were RCTs: seven with a crossover design [35, 36, 49, 51, 54–56], five with a two-arm parallel design [32, 34, 48, 50, 53], and one with a three-arm parallel design [33]. Only one study was a non-randomized controlled crossover trial [52].

The type of cardiac rhythm evaluated in the 14 studies was: (i) mixed (both shockable and non-shockable rhythm) (*n* = 6) [34–36, 52, 54, 55]; (ii) shockable rhythm [ventricular fibrillation (VF)/pulseless ventricular tachycardia (pVT)] (*n* = 6) [33, 48, 50, 51, 53, 56]; (iii) pulseless electrical activity (PEA) (*n* = 1) [49]; (iv) and PEA that changed into VF (*n* = 1) [32].

Teams consisted of medical residents in eight studies [32, 33, 35, 50, 51, 53–55], registered nurses in six [34, 35, 48, 52, 54, 56], attending physicians in six [34, 35, 48, 49, 54, 56], nursing students in one [52] and medical students in another one [36].

Ten studies evaluated the effectiveness of interactive digital cognitive tools installed on an electronic device: four smartphone apps [32, 33, 51, 55], a tablet app [48], an electronic decision support system controlled by a handheld tablet device [52], a dynamic electronic cognitive aid on a large screen display [53], an interactive web-based application intended to be viewed by the entire team on a large screen [54], a computer-based prompting device [50], and a Decision Support Tool on Ipod Touch [36]. Four studies [34, 35, 49, 56] evaluated paper-based cognitive tools. Two studies considered the use of paper-based cognitive aids as a control group [33, 53], including the three-arm RCT [33] that compared a smartphone app with both a paper-based cognitive aid and no support.

Overall, of the 688 IHCA scenarios included in the selected studies, 235 involved the use of an electronic support, 135 a paper-based support and 318 no support. The number of scenarios per study ranged from eight [51, 52] to 53 [55] per arm.

### Quality assessment

The summary assessment of risk of bias of the included RCTs is displayed in Table 2. The only non-randomized controlled trial [52], as inherently lower in quality than RCTs, was not evaluated. All but two studies were at high risk of bias in one or more domains, with the “other sources of bias” domain more frequently classified as high risk. There were no studies at low risk of bias, as the two studies that were not classified as high risk, both had an unclear risk of bias in one or two domains.

**Table 2:**
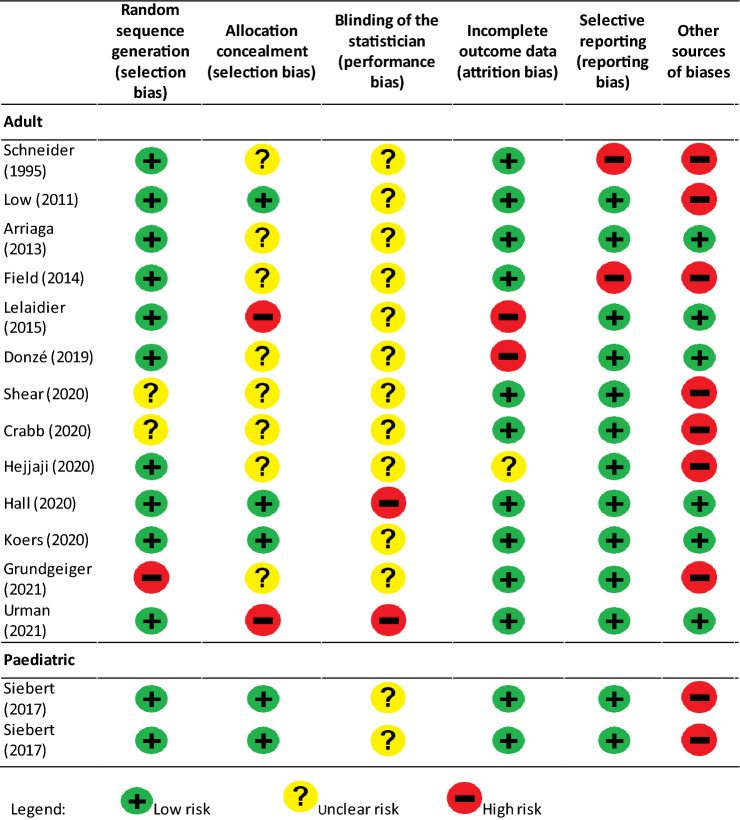
Quality assessment of included studies

### Synthesis of results and meta-analyses

The results for our review of primary and secondary outcomes assessed by the included studies are presented in the supplementary material (Supplementary file 3 and 4).

**Fig. 2:**
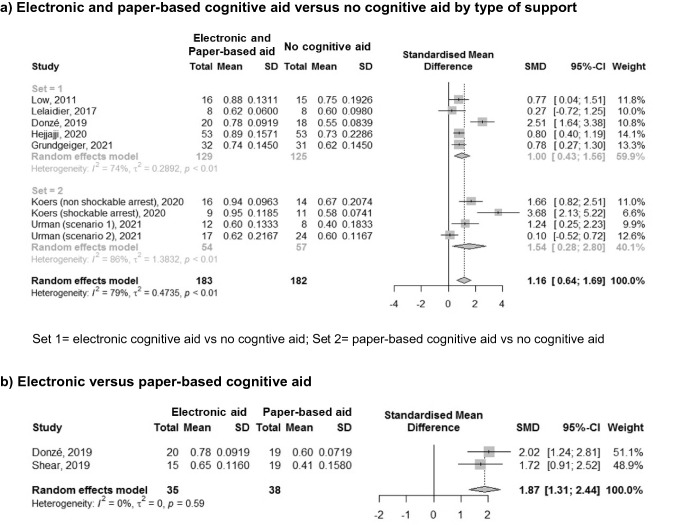
Meta-analyses of adult studies for the outcome of teams’ overall performance

Eleven studies evaluated teams’ overall CA management. Three of them were excluded from the meta-analyses since they did not report a measure of central tendency (mean or median) and/or dispersion of data (standard deviation or interquartile range) in their results [35, 36, 54]. The meta-analyses conducted on the remaining eight studies [32–34, 48, 49, 51, 53, 55] showed that overall performance was significantly better in the groups that used an electronic support tool compared to the groups without any support (Fig. 2a; SMD 1.00, 95% CI 0.43;1.56, *I*^2^ = 74%), in the groups that used a paper-based cognitive aid compared to the group that had no support (Fig. 2a; SMD 1.54, 95% CI 0.28;2.80, *I*^2^ = 86%), and in the groups with an electronic or paper support tool in comparison to the groups that had no support (Fig. 2a; SMD 1.16, 95% CI 0.64; 1.69, *I*^2^ = 79%). Lastly, overall performance was significantly better in the groups that used an electronic cognitive aid in comparison to the groups that used a paper-based cognitive aid (Fig. 2b; SMD 1.87, 95% CI 1.31;2.44, *I*^2^ = 0%). As shown in Fig. 2, the studies by Koers [34] and Urman [49] are reported twice because they analysed and reported the results of two different CA scenarios separately, thus meta-analyses were conducted considering each scenario as an independent analysis unit. In addition, the study by Donzé et al. [33] is included in two meta-analyses (Figs. 2a, b) because this is a three-parallel-arm study that evaluated both electronic and paper-based cognitive aid versus no cognitive aid and electronic versus paper-based cognitive aid. Funnel plots for each meta-analysis are reported in the supplementary material (Supplementary file 5).

Remarkably, among all studies evaluating performance, only two [32, 36] used a previously validated tool (Supplementary file 3). Eight studies [36, 48, 50, 52–56] evaluated deviations from published guidelines, with approximately 50 different deviations being assessed, mostly omissions of critical actions (Supplementary file 4). Overall, fewer deviations were detected in the intervention groups. In particular, two studies evaluated the impact of electronic cognitive aids [52, 53] on failure to defibrillate and failure to administer adrenaline, and reported a significant reduction of these errors in the intervention groups using the electronic tool (Supplementary file 4).

Times to critical actions were analysed by three studies [36, 48, 54]. Two studies [36, 48] assessed the time to first defibrillation; no statistically significant difference was found between groups by Field et al. [36], while a shorter time was reported in the intervention group by Grundgeiger et al. [48], but a statistical comparison was not performed for this specific item (Supplementary file 3). Crabb et al. [54] analysed time differences from guideline recommended rhythm check time (every 2 min), defibrillation time (every 2 min) and adrenaline administration time (every 3 min). Only rhythm check time difference and the aggregate recommended intervention time difference were significantly lower in the intervention group (Supplementary file 3). Grundgeiger et al. [48] analysed time differences from guideline recommended chest compressor change and rhythm check finding lower times to achieve these actions in the intervention group, but the analysis was only descriptive and statistical significance was not reported.

In the only two studies assessing the quality of CPR, [48, 55] Hejjaji et al. [55] reported that the use of the smartphone app (Redivus Code Blue) resulted in a marginally, yet statistically significant, higher CC fraction, while in the study by Grundgeiger et al. [48], which used a different tablet app, a statistical comparison between the intervention and the control group was not performed for CPR metrics (Supplementary file 3).

The non-technical skills of the teams were assessed by three studies [33, 51, 53] which all used validated scores to assess this domain (Supplementary file 3). In the two studies evaluating the same smartphone app (MAX) [33, 51], a significant benefit of the app in improving non-technical skills of the teams was found. However, one study [41] did not report the scores divided by scenario, but only a combined overall score for all five types of scenarios of which only one was a CA case.

Only one study assessed the workload perceived by the participants [48] showing statistically significant lower team leaders’ mental and physical demand, and effort to achieve their performance in the intervention group respect to the control group.

Eight studies [32, 34, 35, 49, 50, 54–56] evaluated the cognitive aid usability. The cognitive supports, both electronic and paper-based, were rated as 'easy to use' in all studies [32, 34, 35] evaluating this domain. Additionally, five out of five studies [32, 34, 35, 54, 56] reported that the majority of health professionals would want to use the cognitive support tool in future real emergencies. Furthermore, most of the participants involved in three studies evaluating this aspect, reported they would like health professionals to use the cognitive support tool if they were themselves the victims of a CA [34, 54, 56].

Potential harm from the use of the cognitive aids under evaluation was also investigated by the included studies. One study [53] found more interruptions during compressions in the intervention group, while two studies [52, 55] highlighted how the use of the cognitive aid could be a source of distraction. Low et al. [32] reported that half of the participants feared a possible perception of non-professionalism by the patient or other health professionals, while Jones et al. [52] reported possible side effects related to the use of an electronic decision system such as unsafe shock delivering and drug administration delays.

## Paediatric studies

### Study characteristics

The characteristics of the two included paediatric studies [30, 31] are reported in Table 1. Both were recent RCTs conducted by the same research team and evaluated pVT scenarios managed by paediatric residents. They both tested an app-format cognitive support tool, one for tablets and one for augmented reality glasses (Google Glasses), compared to a paper-based cognitive aid (PALS pocket reference cards).

### Quality assessment

Overall, the quality assessment showed a low risk of bias for most domains, with the exception of the "statistician blinding" domain, which was not reported by the two studies, and the "other risk of bias" domain which was rated as 'high' for both studies, due to the lack of publication of the study protocols (Table 2).

### Synthesis of results and meta-analyses

**Fig. 3:**
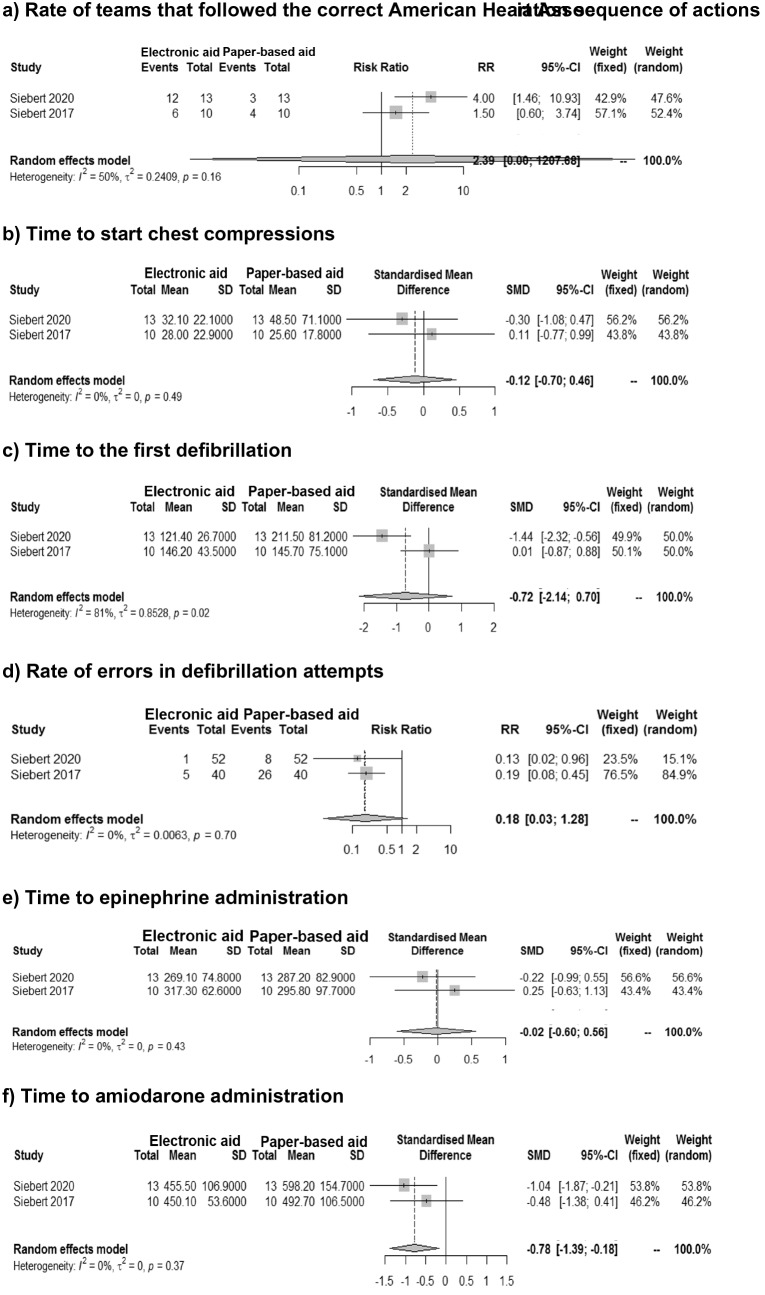
Meta-analyses of outcomes for paediatric studies

Statistically significant improvements of the intervention compared to the control groups were detected only for time to amiodarone administration (SMD − 0.78, 95% CI − 1.39; − 0.18, *I*^2^ = 0%) (Fig. 3). A non-statistically significant improvement in the teams that used the electronic cognitive aid respect to the teams that used the paper-based cognitive aid was detected in time to defibrillation and start of CC, rate of errors in defibrillation attempts, and rate of teams that followed the correct sequence of actions, while no difference was shown in time to administer epinephrine (Fig. 3, Supplementary file 6).

With respect to the stress perceived by participants post scenario, this was significantly lower in the groups that used the electronic cognitive tools, compared with the control groups (Supplementary file 6; SMD − 0.72; 95% CI − 1.32; − 0.11; *I*^2^ = 0%).

The results of the additional outcome measures that were obtained in each of the two paediatric studies, and that could not be combined in meta-analyses, are reported in Supplementary file 7. Neither studies assessed the quality of CPR, the participants’ non-technical skills, and the usability of the cognitive aid.

## Discussion

Our findings show that the use of cognitive aids appears to reduce deviations from guideline recommendations and improve overall resuscitation performance in the management of simulated IHCA scenarios. While the overall use of either a digital or paper-based cognitive aid is associated with an improvement of the assessed outcomes, the number and/or the heterogeneity of the included studies with respect to the outcome(s) assessed, the intervention and the comparator, substantially limited our ability to pool their results in a robust meta-analysis. Studies assessing the effectiveness of using cognitive aids for the management of IHCA in the simulation setting are still scant, especially in paediatrics, and the comparison of different types of cognitive aids limited. Only one of the 16 included studies endeavoured to compare a digital aid, versus a paper aid, versus no cognitive aid, while only three studies (2 paediatric and one adult) compared a digital versus a paper-based cognitive aid. Although the digital tools showed to be superior to the paper cognitive aids in all these studies, the evidence is not sufficient to recommend digital over paper-based cognitive aids, especially considering the heterogeneity between studies and individual study limitations.

While the past five years have seen an increase in interest in this field, with most of the studies published in this time frame, this is not yet on par with the literature about the use of cognitive aids for the management of OHCA, for which several studies and systematic reviews have already been published [39, 41, 57–60]. Notably, a main difference in the types of cognitive tools employed in OHCA and IHCA is that cognitive aids for OHCA are mostly designed to support lay rescuers to assist a victim of CA and are primarily focussed on asking for help and on the quality of CPR [39, 41, 58–60]. Thus, findings cannot be generalized across the two settings. The relative lack of studies investigating cognitive support aids for IHCA is also underlined by a recent systematic review about cognitive aid use during resuscitation, published by the ILCOR [42], which could not find any study assessing the management of IHCA real-life scenarios by healthcare professionals, except for one article [61]. This article was however excluded from the current review, since the examined cognitive aid did not support the overall management of CA, but only single aspects of resuscitation (i.e. clarifying roles and team coordination).

To the best of our knowledge, this review is the first to systematically evaluate the effectiveness of cognitive aids in helping healthcare professionals optimize the management of adult or paediatric simulated IHCA. Our study shows that cognitive aids improved overall performance and adherence to guidelines, especially in terms of fewer omission of actions, such as failure to defibrillate and failure to administer adrenaline for adult CA, and in terms of decreased errors (mainly related to defibrillation) for paediatric CA. These findings are clinically relevant because a greater number of deviations from guidelines have previously been associated with a lower probability of achieving Return Of Spontaneous Circulation (ROSC) and survival to hospital discharge [62, 63].

The times of critical actions also resulted to be shorter in the intervention groups. The reduction in time to perform interventions is also clinically relevant, as different studies report that a shorter time to interventions is associated with better clinical outcomes [16, 22, 64–67]. Moreover, in one study that evaluated the quality of CPR, the compression fraction was higher with the use of the cognitive aid; this is an essential component of resuscitation as it could independently predict a higher survival rate [68, 69].

The non-technical skills within the intervention teams were rated better than those of the control teams. An improvement in non-technical skills may result in improved overall resuscitation and patient outcome [70, 71].

Cognitive aids assessed for usability received positive reviews. However, concerns were raised regarding the possible risk for distraction, reduction of situational awareness and perception of unprofessionalism. For example, the use of the cognitive aid was associated with more interruptions during CPR in one study [53] and this could in turn be associated with a reduction in victim survival in real-life scenarios [72]. Moreover, it is important to consider that the use of cognitive support tools could increase users’ cognitive load. Remarkably, in the current meta-analysis, the perceived workload resulted to be significantly reduced in the app groups. A reduction of 'workload' can improve the performance of the resuscitation team [73]. Possible adverse effects must always be considered to refine cognitive tools and optimize users’ interaction with them, to avoid a negative influence on clinical outcomes.

The quality assessment of the included studies showed a very heterogeneous risk of bias. The paediatric studies received overall good evaluations; the risk of bias was linked to the absence of published protocols and to the unspecified "blinding" of the statistician. Adult studies showed a medium–high risk of bias linked not only to the lack of report on the “blinding” of the statistician and allocation concealment processes, but also to the absence of a published protocol. Only two studies [35, 56], evaluating paper cognitive aids, were not classified as high risk, but both had an unclear risk of bias in one or two domains. Furthermore, it is important to highlight that the number of scenarios per study arm was limited in most of the studies.

Finally, a limitation of this systematic review is the inclusion of studies conducted in the simulation setting. We focussed on simulation studies because, according to a recent systematic review conducted by the ILCOR group [42], no study at the time of the review had evaluated the impact of cognitive aids on the management of real-life CA. Additionally, as we limited our search to healthcare databases, possible relevant literature from the field of usability, human factor design or human computer interaction may have possibly been excluded. However, the use of a highly sensitive search strategy is very likely to have identified all relevant studies, limiting the number of studies that might have been missed. Furthermore, we included in the meta-analyses RCTs with a crossover design as if they were parallel group RCTs and this approach might have affected our results, given the learning effect related to the former study design. The three-adult crossover RCTs [49, 51, 55] included in the meta-analyses found non-significant or only mild improvement in the group using a cognitive aid, thus leading to potential underestimation of the benefit of using cognitive support tools. Lastly, we had to exclude ten potentially relevant records (0.24% of excluded studies) as we could not obtain complete information in the methods and results from contacting the authors.

Considering the findings of this systematic review, it is advisable that future studies follow the extensions of the CONSORT and STROBE Statements [74] for simulation-based research to guarantee a rigorous study methodology and comparable results. Given the high heterogeneity of included studies, especially in terms of outcome measures, we also suggest that future studies use the same validated tools for the assessment of their outcomes (i.e. team performance, non-technical skills, workload). As for the definition of time to perform critical interventions, the focus on clinically relevant actions for resuscitation and the use of clearly defined timeframes (i.e. from the recognition of cardiac arrest to the specific action analysed), will allow a correct interpretation of the results, the comparisons between different studies, and robust meta-analyses in the future.

Furthermore, as already pointed out by Marshall et al. [75], most studies on cognitive aids focus on content and less on product design, presentation and usability. A review performed by Metelman et al. [40] showed that most of the apps available in online stores for the management of OHCA are not tested for content, effectiveness and usability. Therefore, since cognitive aids should be developed based on users’ needs to be easy to use and intuitive to interact with, as reported in some promising experience [76–78], we strongly encourage that future studies assess the usability of cognitive aids employed for IHCA management with validated and reliable tools, such as the System Usability Scale [79].

## Conclusions

Although published studies are scant and heterogeneous, the use of cognitive aids appears promising in reducing deviations from guideline recommendations in the management of simulated adult IHCA scenarios, with potential positive impact on clinical practice. As for paediatric studies, the low number of studies and scenarios included, without a control group using no cognitive aid, does not allow to conclude in favour or against the use of cognitive aids. Further studies using a rigorous methodology and comparable outcome measures will help provide a definitive answer on the effectiveness of cognitive aids in improving health professionals’ management of IHCA.

## Supplementary Information

Below is the link to the electronic supplementary material.

Supplementary file1 (DOCX 27 KB)

Supplementary file2 (DOCX 16 KB)

Supplementary file3 (DOCX 31 KB)

Supplementary file4 (DOCX 24 KB)

Supplementary file5 (DOCX 116 KB)

Supplementary file6 (DOCX 3111 KB)

Supplementary file7 (DOCX 26 KB)

## Data Availability

All data generated or analysed during this study are included in this published article (and its supplementary information files).
