# Supplementary material for: The impact of cognitive aids on resuscitation performance in in-hospital cardiac arrest scenarios: a systematic review and meta-analysis
Source: Intern Emerg Med. 2022 Aug 29;17(7):2143–58. doi: 10.1007/s11739-022-03041-6 (PMC9420676; doi:10.1007/s11739-022-03041-6)
Supplement: Supplementary file 1 — Supplementary file1 (DOCX 27 KB) [file 11739_2022_3041_MOESM1_ESM.docx]

**The impact of cognitive aids on resuscitation performance in simulated in-hospital cardiac arrest scenarios: a systematic review and meta-analysis**

**Supplementary file 1. Search strategies (1^st^ January 1974 – 31^st^ December 2021)**

**MEDLINE/PubMed**

**1) Cardiopulmonary resuscitation**

#1 "Heart Arrest"[Mesh:NoExp] OR "Resuscitation"[Mesh:NoExp] OR "Cardiopulmonary Resuscitation"[Mesh] OR "Death, Sudden, Cardiac"[Mesh:NoExp] OR "Life Support Care"[Mesh:NoExp] OR "Ventricular Fibrillation"[Mesh] OR "Heart Massage"[Mesh]

#2 "Heart arrest" OR “Cardiac arrest*” OR “Cardiopulmonary arrest*” OR “Cardio-pulmonary arrest” OR "Sudden cardiac death*" OR “Cardiac Sudden Death” OR "Cardiovascular arrest" OR Asystol* OR "Pulseless electrical activity" OR “Ventricular fibrillation*” OR "Electromechanical Dissociation" OR "CPR" OR Resuscitation* OR "Heart massage" OR "Cardiac massage" OR "Code blue" OR "Cardiac life support" OR "Life support care" OR “cardiorespiratory arrest*” OR “cardio-respiratory arrest”

#1 OR #2

**2) Cognitive Aid**

#3 "Mobile Applications"[Mesh] OR "Software"[MeSH:NoExp] OR "Computers, Handheld"[Mesh] OR "Cell Phone"[Mesh:NoExp] OR "Augmented Reality"[Mesh] OR "Decision Support Systems, Clinical"[Mesh] OR "Checklist"[Mesh] OR "Pamphlets"[Mesh] OR "Decision Support Techniques"[Mesh:NoExp] OR "Software Design"[Mesh] OR "Audiovisual Aids"[Mesh]

#4 “App” OR “Apps” OR Application* OR Mobile OR “Smartphone*” OR “Smart-phone*” OR “phone*” OR “telephone*” OR “Software*” OR “decision support*” OR “decision tool*” OR “support tool*” OR “Cognitive aid” OR “cognitive aids” OR Tablet OR “Hand-held” OR “Handheld” OR “Smart device*” OR “Electronic tool*” OR “computer program*” OR “Digital Assistant*” OR “Google glass*” OR “Augmented reality” OR “Decision-Making Aid” OR “Decision-Making Aids” OR “Decision aid” OR “decision aids” OR “poster” OR “posters” OR checklist* OR pamphlet* OR booklet* OR notebook OR “pocket book*” OR “picture” OR video OR flowchart OR “flow chart” OR eHealth OR "audiovisual aid" OR "audiovisual aids" OR "visual aid" OR "visual aids"

#3 OR #4

**3) Simulation**

#5 "Simulation Training"[Mesh]

#6 simulat* OR scenario* OR manikin* OR mannequin* OR “mock code” OR “mechanical model”

#5 OR #6

**4) Cardiopulmonary resuscitation & Cognitive Aid & Simulation**

(#1 OR #2) AND (#3 OR #4) AND (#5 OR #6)

((("Heart Arrest"[Mesh:NoExp] OR "Resuscitation"[Mesh:NoExp] OR "Cardiopulmonary Resuscitation"[Mesh] OR "Death, Sudden, Cardiac"[Mesh:NoExp] OR "Life Support Care"[Mesh:NoExp] OR "Ventricular Fibrillation"[Mesh] OR "Heart Massage"[Mesh]) OR ("Heart arrest" OR “Cardiac arrest*” OR “Cardiopulmonary arrest*” OR “Cardio-pulmonary arrest” OR "Sudden cardiac death*" OR “Cardiac Sudden Death” OR "Cardiovascular arrest" OR Asystol* OR "Pulseless electrical activity" OR “Ventricular fibrillation*” OR "Electromechanical Dissociation" OR "CPR" OR Resuscitation* OR "Heart massage" OR "Cardiac massage" OR "Code blue" OR "Cardiac life support" OR "Life support care" OR “cardiorespiratory arrest*” OR “cardio-respiratory arrest”)) AND (("Mobile Applications"[Mesh] OR "Software"[MeSH:NoExp] OR "Computers, Handheld"[Mesh] OR "Cell Phone"[Mesh:NoExp] OR "Augmented Reality"[Mesh] OR "Decision Support Systems, Clinical"[Mesh] OR "Checklist"[Mesh] OR "Pamphlets"[Mesh] OR "Decision Support Techniques"[Mesh:NoExp] OR "Software Design"[Mesh] OR "Audiovisual Aids"[Mesh]) OR (“App” OR “Apps” OR Application* OR Mobile OR “Smartphone*” OR “Smart-phone*” OR “phone*” OR “telephone*” OR “Software*” OR “decision support*” OR “decision tool*” OR “support tool*” OR “Cognitive aid” OR “cognitive aids” OR Tablet OR “Hand-held” OR “Handheld” OR “Smart device*” OR “Electronic tool*” OR “computer program*” OR “Digital Assistant*” OR “Google glass*” OR “Augmented reality” OR “Decision-Making Aid” OR “Decision-Making Aids” OR “Decision aid” OR “decision aids” OR “poster” OR “posters” OR checklist* OR pamphlet* OR booklet* OR notebook OR “pocket book*” OR “picture” OR video OR flowchart OR “flow chart” OR eHealth OR "audiovisual aid" OR "audiovisual aids" OR "visual aid" OR "visual aids"))) AND (("Simulation Training"[Mesh]) OR (simulat* OR scenario* OR manikin* OR mannequin* OR “mock code” OR “mechanical model”))

**CINAHL Complete**

1. **Cardiopulmonary resuscitation**

#1 (MH "Heart Arrest+") OR (MH "Resuscitation, Cardiopulmonary+") OR (MH "Resuscitation") OR (MH "Heart Massage") OR (MH "Ventricular Fibrillation") OR (MH "Death, Sudden, Cardiac")

#2 "Heart arrest*" OR "Cardi* arrest*" OR "Sudden cardiac death*" OR “Cardiac sudden death” OR "Asystol*" OR "Pulseless electrical activity" OR "Ventricular fibrillation*" OR "Electromechanical Dissociation" OR "CPR" OR "Resuscitation*" OR "Heart massage" OR "Cardiac massage" OR "Code blue" OR "Cardiac life support" OR "Life support care"

#1 OR #2

1. **Cognitive aid**

#3 (MH "Mobile Applications") OR (MH "Software") OR (MH "Computers, Portable+") OR (MH "Cellular Phone") OR (MH "Augmented Reality") OR (MH "Decision Support Systems, Clinical") OR (MH "Decision Support Techniques") OR (MH "Checklists") OR (MH "Pamphlets") OR (MH "Software Design")

#4 (“App” OR “Apps” OR Application* OR Mobile OR “Smartphone*” OR “Smart-phone*” OR “phone*” OR OR “telephone*” OR “Software*” OR “decision support*” OR “decision tool*” OR “support tool*” OR “Cognitive aid*” OR Tablet OR “Hand-held” OR “Handheld” OR “Smart device*” OR “Electronic tool*” OR “computer program*” OR “Digital Assistant*” OR “Google glass*” OR “Augmented reality” OR “Decision-Making Aid*” OR “Decision aid*” OR “poster” OR “posters” OR checklist* OR pamphlet* OR booklet* OR notebook OR “pocket book*” OR “picture” OR video OR flowchart OR “flow chart” OR eHealth OR "audiovisual aid*" OR "visual aid*")

#3 OR #4

1. **Simulation**

#5 (MH "Simulations") OR (MH "Patient Simulation")

#6 simulat* OR scenario* OR manikin* OR mannequin* OR “mock code” OR “mechanical model”

#5 or #6

1. **Cardiopulmonary resuscitation & Cognitive Aid & Simulation**

(#1 OR #2) AND (3# OR #4) AND (#5 OR #6)

( ( (MH "Heart Arrest+") OR (MH "Resuscitation, Cardiopulmonary+") OR (MH "Resuscitation") OR (MH "Heart Massage") OR (MH "Ventricular Fibrillation") OR (MH "Death, Sudden, Cardiac") ) OR ( "Heart arrest*" OR "Cardi* arrest*" OR "Sudden cardiac death*" OR “Cardiac sudden death” OR "Asystol*" OR "Pulseless electrical activity" OR "Ventricular fibrillation*" OR "Electromechanical Dissociation" OR "CPR" OR "Resuscitation*" OR "Heart massage" OR "Cardiac massage" OR "Code blue" OR "Cardiac life support" OR "Life support care") ) AND ( ( (MH "Mobile Applications") OR (MH "Software") OR (MH "Computers, Portable+") OR (MH "Cellular Phone") OR (MH "Augmented Reality") OR (MH "Decision Support Systems, Clinical") OR (MH "Decision Support Techniques") OR (MH "Checklists") OR (MH "Pamphlets") OR (MH "Software Design")) OR (“App” OR “Apps” OR Application* OR Mobile OR “Smartphone*” OR “Smart-phone*” OR “ phone*” OR “ telephone*” OR “Software*” OR “decision support*” OR “decision tool*” OR “support tool*” OR “Cognitive aid*” OR Tablet OR “Hand-held” OR “Handheld” OR “Smart device*” OR “Electronic tool*” OR “computer program*” OR “Digital Assistant*” OR “Google glass*” OR “Augmented reality” OR “Decision-Making Aid*” OR “Decision aid*” OR “poster” OR “posters” OR checklist* OR pamphlet* OR booklet* OR notebook OR “pocket book*” OR “picture” OR video OR flowchart OR “flow chart” OR eHealth OR "audiovisual aid*" OR "visual aid*") ) AND ( ((MH "Simulations") OR (MH "Patient Simulation")) OR (simulat* OR scenario* OR manikin* OR mannequin* OR “mock code” OR “mechanical model”) )

**EMBASE**

Until 31^st^ May 2020

**1) Cardiopulmonary resuscitation**

#1. 'heart arrest'/de OR 'cardiopulmonary arrest'/exp OR 'sudden cardiac death'/exp OR 'resuscitation'/exp OR 'heart massage'/exp OR 'heart ventricle fibrillation'/de

#2. ('heart arrest' OR 'cardi* arrest*' OR 'cardio-pulmonary arrest' OR 'cardio-respiratory arrest' OR 'sudden cardiac death*' OR ‘Cardiac sudden death’ OR asystol* OR 'pulseless electrical activity' OR ‘ventricular fibrillation*’ OR 'electromechanical dissociation' OR 'cpr' OR 'resuscitation*' OR 'heart massage' OR 'cardiac massage' OR 'code blue' OR 'cardiac life support' OR 'life support care'):ti,ab,kw

#3. #1 OR #2

**2) Cognitive Aid**

#4. 'mobile application'/exp OR 'healthcare software'/exp OR 'personal digital assistant'/exp OR 'tablet computer'/exp OR 'mobile phone'/exp OR 'electronic device'/de OR 'augmented reality'/exp OR 'decision aid'/exp OR 'decision support system'/de OR 'clinical decision support system'/exp OR 'cognitive aid'/exp OR 'checklist'/exp OR 'software design'/exp OR 'audiovisual aid'/exp

#5. ('app' OR 'apps' OR application* OR mobile OR 'smartphone*' OR 'smart-phone*' OR 'phone*' OR 'telephone*' OR 'software*' OR 'decision support*' OR 'decision tool*' OR 'support tool*' OR 'cognitive aid*' OR tablet OR 'hand-held' OR 'handheld' OR 'smart device*' OR 'electronic tool*' OR 'computer program*' OR 'digital assistant*' OR 'google glass*' OR 'augmented reality' OR 'decision-making aid*' OR 'decision aid*' OR 'poster' OR 'posters' OR checklist* OR pamphlet* OR booklet* OR notebook OR 'pocket book*' OR 'picture' OR video OR flowchart OR 'flow chart' OR ehealth OR 'audiovisual aid*' OR 'visual aid*'):ti,ab,kw

#6. #4 OR #5

**3) Simulation**

#7 'simulation'/de OR 'high-fidelity simulation'/exp OR 'patient simulation'/exp OR 'manikin'/de

#8 (simulat* OR scenario* OR manikin* OR mannequin* OR 'mock code' OR 'mechanical model'):ti,ab,kw

#9 #7 OR #8

**4) Cardiopulmonary resuscitation & Cognitive Aid & Simulation**

#10 #3 AND #6 AND #9

('heart arrest'/de OR 'cardiopulmonary arrest'/exp OR 'sudden cardiac death'/exp OR 'resuscitation'/exp OR 'heart massage'/exp OR 'heart ventricle fibrillation'/de OR ‘heart arrest':ti,ab,kw OR 'cardi* arrest*':ti,ab,kw OR 'cardio-pulmonary arrest':ti,ab,kw OR 'cardio-respiratory arrest':ti,ab,kw OR 'sudden cardiac death*':ti,ab,kw OR 'cardiac sudden death':ti,ab,kw OR asystol*:ti,ab,kw OR 'pulseless electrical activity':ti,ab,kw OR 'ventricular fibrillation*':ti,ab,kw OR 'electromechanical dissociation':ti,ab,kw OR 'cpr':ti,ab,kw OR 'resuscitation*':ti,ab,kw OR 'heart massage':ti,ab,kw OR 'cardiac massage':ti,ab,kw OR 'code blue':ti,ab,kw OR 'cardiac life support':ti,ab,kw OR 'life support care':ti,ab,kw) AND ('mobile application'/exp OR 'healthcare software'/exp OR 'personal digital assistant'/exp OR 'tablet computer'/exp OR 'mobile phone'/exp OR 'electronic device'/de OR 'augmented reality'/exp OR 'decision aid'/exp OR 'decision support system'/de OR 'clinical decision support system'/exp OR 'cognitive aid'/exp OR 'checklist'/exp OR 'software design'/exp OR 'audiovisual aid'/exp OR 'app':ti,ab,kw OR 'apps':ti,ab,kw OR application*:ti,ab,kw OR mobile:ti,ab,kw OR 'smartphone*':ti,ab,kw OR 'smart-phone*':ti,ab,kw OR 'phone*':ti,ab,kw OR 'telephone*':ti,ab,kw OR 'software*':ti,ab,kw OR 'decision support*':ti,ab,kw OR 'decision tool*':ti,ab,kw OR 'support tool*':ti,ab,kw OR 'cognitive aid*':ti,ab,kw OR tablet:ti,ab,kw OR 'hand-held':ti,ab,kw OR 'handheld':ti,ab,kw OR 'smart device*':ti,ab,kw OR 'electronic tool*':ti,ab,kw OR 'computer program*':ti,ab,kw OR 'digital assistant*':ti,ab,kw OR 'google glass*':ti,ab,kw OR 'augmented reality':ti,ab,kw OR 'decision-making aid*':ti,ab,kw OR 'decision aid*':ti,ab,kw OR 'poster':ti,ab,kw OR 'posters':ti,ab,kw OR checklist*:ti,ab,kw OR pamphlet*:ti,ab,kw OR booklet*:ti,ab,kw OR notebook:ti,ab,kw OR 'pocket book*':ti,ab,kw OR 'picture':ti,ab,kw OR video:ti,ab,kw OR flowchart:ti,ab,kw OR 'flow chart':ti,ab,kw OR ehealth:ti,ab,kw OR 'audiovisual aid*':ti,ab,kw OR 'visual aid*':ti,ab,kw) AND ('simulation'/de OR 'manikin'/de OR 'high-fidelity simulation'/exp OR 'patient simulation'/exp OR simulat*:ti,ab,kw OR scenario*:ti,ab,kw OR manikin*:ti,ab,kw OR mannequin*:ti,ab,kw OR 'mock code':ti,ab,kw OR 'mechanical model':ti,ab,kw)

From 31^st^ May 2020, until 31^st^ December 2021

1 ('heart arrest' or 'cardi* arrest*' or 'cardio-pulmonary arrest' or 'cardio-respiratory arrest' or 'sudden cardiac death*' or 'cardiac sudden death' or 'asystol*' or 'pulseless electrical activity' or 'ventricular fibrillation*' or 'electromechanical dissociation' or 'cpr' or 'resuscitation*' or 'heart massage' or 'cardiac massage' or 'code blue' or 'cardiac life support' or 'life support care').ab,kw,ti. 190394

2 ('app' or 'apps' or application* or mobile or 'smartphone*' or 'smart-phone*' or 'phone*' or 'telephone*' or 'software*' or 'decision support*' or 'decision tool*' or 'support tool*' or 'cognitive aid*' or tablet or 'hand-held' or 'handheld' or 'smart device*' or 'electronic tool*' or 'computer program*' or 'digital assistant*' or 'google glass*' or 'augmented reality' or 'decision-making aid*' or 'decision aid*' or 'poster' or 'posters' or checklist* or pamphlet* or booklet* or notebook or 'pocket book*' or 'picture' or video or flowchart or 'flow chart' or 'telemedicine' or telehealth or ehealth or 'audiovisual aid*' or 'visual aid*').ab,kw,ti. 2651936

3 (simulat* or scenario* or manikin* or mannequin* or 'mock code' or 'mechanical model').ab,kw,ti. 787612

4 *heart arrest/ 25824

5 exp cardiopulmonary arrest/ 6061

6 exp sudden cardiac death/ 19433

7 exp resuscitation/ 120772

8 exp heart massage/ 2330

9 *heart ventricle fibrillation/ 10822

10 4 or 5 or 6 or 7 or 8 or 9 163603

11 1 or 10 251269

12 exp mobile application/ 18765

13 exp healthcare software/ 2618

14 exp personal digital assistant/ 1676

15 exp tablet computer/ 2250

16 exp mobile phone/ 38040

17 exp augmented reality/ 1178

18 exp clinical decision support system/ 4396

19 exp checklist/ 29388

20 *telehealth/ 5711

21 *telemedicine/ 18769

22 exp audiovisual aid/ 950

23 *decision support system/ 11398

24 exp software design/ 1062

25 *electronic device/ 3309

26 12 or 13 or 14 or 15 or 16 or 17 or 18 or 19 or 20 or 21 or 22 or 23 or 24 or 25 124866

27 2 or 26 2676075

28 *simulation/ 23420

29 exp high-fidelity simulation/ 281

30 exp patient simulation/ 1448

31 *manikin/ 471

32 28 or 29 or 30 or 31 25526

33 3 or 32 788604

34 11 and 27 and 33 2398

35 limit 34 to dc=20200531-20211231 363

**Cochrane Library**

ID Search Hits

#1 MeSH descriptor: [Heart Arrest] this term only

#2 MeSH descriptor: [Death, Sudden, Cardiac] this term only

#3 MeSH descriptor: [Cardiopulmonary Resuscitation] explode all trees

#4 MeSH descriptor: [Resuscitation] this term only

#5 MeSH descriptor: [Ventricular Fibrillation] this term only

#6 MeSH descriptor: [Life Support Care] this term only

#7 MeSH descriptor: [Heart Massage] this term only

#8 (heart NEXT arrest):ti,ab,kw

#9 (cardi* NEXT arrest*):ti,ab,kw

#10 (sudden NEXT cardiac NEXT death*):ti,ab,kw

#11 ("cardiac sudden death"):ti,ab,kw

#12 (asystol*):ti,ab,kw

#13 ("pulseless electrical activity"):ti,ab,kw

#14 (ventricular NEXT fibrillation*):ti,ab,kw

#15 ("electromechanical dissociation"):ti,ab,kw

#16 (CPR):ti,ab,kw

#17 ("resuscitation*"):ti,ab,kw

#18 ("heart massage"):ti,ab,kw

#19 ("cardiac massage"):ti,ab,kw

#20 ("code blue"):ti,ab,kw

#21 ("cardiac life support"):ti,ab,kw

#22 ("life support care"):ti,ab,kw

#23 {OR #1-#22}

#24 MeSH descriptor: [Mobile Applications] this term only

#25 MeSH descriptor: [Computers, Handheld] explode all trees

#26 MeSH descriptor: [Cell Phone] this term only

#27 MeSH descriptor: [Software] this term only

#28 MeSH descriptor: [Augmented Reality] this term only

#29 MeSH descriptor: [Decision Support Systems, Clinical] this term only

#30 MeSH descriptor: [Decision Support Techniques] this term only

#31 MeSH descriptor: [Checklist] this term only

#32 MeSH descriptor: [Pamphlets] this term only

#33 MeSH descriptor: [Telemedicine] this term only

#34 MeSH descriptor: [Software Design] this term only

#35 MeSH descriptor: [Audiovisual Aids] this term only

#36 (Digital NEXT assistant*):ti,ab,kw

#37 (Electronic NEXT tool*):ti,ab,kw

#38 (Decision NEXT tool*):ti,ab,kw

#39 (Mobile):ti,ab,kw

#40 (smart NEXT device*):ti,ab,kw

#41 (application*):ti,ab,kw

#42 (App):ti,ab,kw

#43 (Apps):ti,ab,kw

#44 (smartphone*):ti,ab,kw

#45 (smart-phone*):ti,ab,kw

#46 (phone*):ti,ab,kw

#47 (telephone*):ti,ab,kw

#48 (software*):ti,ab,kw

#49 (computer NEXT program*):ti,ab,kw

#50 (Tablet):ti,ab,kw

#51 ("handheld"):ti,ab,kw

#52 ("Hand held"):ti,ab,kw

#53 (Google NEXT Glass*):ti,ab,kw

#54 ("augmented reality"):ti,ab,kw

#55 (support NEXT tool*):ti,ab,kw

#56 (cognitive NEXT aid*):ti,ab,kw

#57 (decision NEXT support*):ti,ab,kw

#58 (decision NEXT aid*):ti,ab,kw

#59 ("Decision making" NEXT aid*):ti,ab,kw

#60 (poster):ti,ab,kw

#61 (posters):ti,ab,kw

#62 (checklist*):ti,ab,kw

#63 (pamphlet*):ti,ab,kw

#64 (booklet*):ti,ab,kw

#65 (notebook):ti,ab,kw

#66 (pocket NEXT book*):ti,ab,kw

#67 (picture):ti,ab,kw

#68 (video):ti,ab,kw

#69 (flowchart):ti,ab,kw

#70 (flow-chart):ti,ab,kw

#71 (telemedicine):ti,ab,kw

#72 (telehealth):ti,ab,kw

#73 (eHealth):ti,ab,kw

#74 (audiovisual NEXT aid*):ti,ab,kw

#75 (visual NEXT aid*):ti,ab,kw

#76 {OR #24-#75}

#77 MeSH descriptor: [Simulation Training] explode all trees

#78 MeSH descriptor: [Manikins] this term only

#79 (simulat*):ti,ab,kw

#80 (scenario*):ti,ab,kw

#81 (manikin*):ti,ab,kw

#82 (mannequin*):ti,ab,kw

#83 (mock NEXT code):ti,ab,kw

#84 (mechanical NEXT model):ti,ab,kw

#85 {OR #78-#84}

#86 #23 and #76 and #85

**Clinical Trials.gov**

**1. Condition or disease**

“Cardiac arrest” OR "Sudden cardiac death" OR "Ventricular fibrillation" OR Resuscitation OR CPR OR "Cardiac massage" OR "life support"

**2. Intervention/treatment**

Application OR software OR smartphone OR phone OR mobile OR “support tool” OR “decision support” OR “cognitive aid” OR “decision aid” OR video OR tablet OR handheld OR checklist OR flowchart
