# Supplementary material for: The impact of cognitive aids on resuscitation performance in in-hospital cardiac arrest scenarios: a systematic review and meta-analysis
Source: Intern Emerg Med. 2022 Aug 29;17(7):2143–58. doi: 10.1007/s11739-022-03041-6 (PMC9420676; doi:10.1007/s11739-022-03041-6)
Supplement: Supplementary file 2 — Supplementary file2 (DOCX 16 KB) [file 11739_2022_3041_MOESM2_ESM.docx]

**The impact of cognitive aids on resuscitation performance in simulated in-hospital cardiac arrest scenarios: a systematic review and meta-analysis**

**Supplementary file 2. Message indicated by the World Health Organization International Clinical Trials Registry Platform**

“Due to heavy traffic generated by the COVID-19 outbreak, the ICTRP Search Portal is not responding from outside WHO temporarily. A new search platform is needed to be able to cope with the high load. Please subscribe to the ICTRP listserv if you wish to be notified when the search portal is working again. Information on how to subscribe can be found on the same page below.”
