# Supplementary material for: The impact of cognitive aids on resuscitation performance in in-hospital cardiac arrest scenarios: a systematic review and meta-analysis
Source: Intern Emerg Med. 2022 Aug 29;17(7):2143–58. doi: 10.1007/s11739-022-03041-6 (PMC9420676; doi:10.1007/s11739-022-03041-6)
Supplement: Supplementary file 3 — Supplementary file3 (DOCX 31 KB) [file 11739_2022_3041_MOESM3_ESM.docx]

**The impact of cognitive aids on resuscitation performance in simulated in-hospital cardiac arrest scenarios: a systematic review and meta-analysis**

**Supplementary file 3. Summary of adult studies results for the primary and secondary outcomes of our systematic review**

| **Study** | **Primary Outcomes** | **Intervention** | **Control** | ***p*** |
| --- | --- | --- | --- | --- |
|  | **Team performance** |  |  |  |
| Low D, 2011^32^ | CAST test scoring system (max score 96)^a^ | 84.5 (75.5-92.5) | 72.0 (62-87) | **0.02** |
| Arriaga AF, 2013^35^ | % failure to adhere to Critical Steps in ACLS scenarios | 7/100 (7%) | 15/89 (17%) | **0.005** |
| Arriaga AF, 2013^35^ | % failure to adhere to Critical Steps in ACLS scenario preceded by hemodynamically unstable condition | 14/154 (9%) | 46/172 (27%) | **<0.001** |
| Field LC, 2014^36^ | Checklist (McEvoy et al 2012)^c^ | 78.4% (n.a.) | 71.8% (n.a.) | **<0.001** |
| Field LC, 2014^36^ | Checklist (McEvoy et al 2012) Correct management steps^c^ | 84.7% (n.a.) | 73.8% (n.a.) | **<0.001** |
| Field LC, 2014^36^ | Checklist (McEvoy et al 2012) Patient assessment steps^c^ | 73.8% (n.a.) | 70.4% (n.a.) | 0.103 |
| Lelaidier R, 2017^51^ | Score based on SFAR and ESC guidelines^b^ | 62.5% (6.0%) | 60.2% (9.8%) | 0.450 |
| Donzé P, 2019^33^ | Score based on SFAR and ERC guidelines^a,b^ | 80% (72%-84%)^a^  77.5% (9.19%)^b^ | PCA: 61% (55%-65%)^a^, 60.4% (7.19%)^b^  No support: 55% (50%-60%)^a^, 54.9%, (8.39%)^b^ | **0.0002**  **<0.0001** |
| Shear TD, 2019^53^ | Checklist performance score (0-5) ^b^ | 3.27 (0.58) | 2.03 (0.79) | **0.0001** |
| Crabb DB, 2020^54^ | % of ACLS recommended interventions delivered  within 60 s | 94% | 91% | 0.30 |
| Crabb DB, 2020^54^ | % of ACLS recommended interventions delivered within 30 s | 90% | 81% | **0.045** |
| Hejjaji V, 2020^55^ | Number of correct ACLS interventions (0-7) ^b^ | 6.2 (1.1) | 5.1 (1.6) | **<0.001** |
| Koers L, 2020^34^ | % of omitted critical management steps*****: shockable CA^a^ | 5% (0-16%) | 42% (37%-47%) | **0.010** |
| Koers L, 2020^34^ | % of omitted critical management steps*****: non-shockable CA^a^ | 6% (0-13%) | 33% (22%-50%) | **0.000** |
| Grundgeiger T, 2021^48^ | CPR performance score (0-2)^b^ | 1.47 (0.29) | 1.24 (0.29) | **0.002** |
| Urman RD, 2021^49^ | Team performance score of CA management: scenario 1 (1-7) ^b^ | 4.6 (0.8) | 3.4 (1.1) | **0.004** |
| Urman RD, 2021^49^ | Team performance score of CA management: scenario 2 (1-7) ^b^ | 4.7 (1.3) | 4.6 (0.7) | **0.530** |
|  | **Time to perform resuscitation actions** |  |  |  |
| Grundgeiger, 2021^48^ | Time to first rhythm analysis (s) | 74 (30) | 98 (53) | n.a. |
| Field LC, 2014^36^ | Time of first shock (s) | 63.6 (n.a.) | 65.7 (n.a.) | 0.808 |
| Grundgeiger, 2021^48^ | Time of first shock (s) | 111 (67) | 135 (82) | n.a. |
|  | **Delays in time to perform resuscitation actions** |  |  |  |
| Jones I, 2019^52^ | Delayed shock | 0/8 | 1/8 | n.a. |
| Jones I, 2019^52^ | Delayed adrenaline | 2/8 | 1/8 | n.a. |
| Jones I, 2019^52^ | Delayed amiodarone | 2/8 | 1/8 | n.a. |
| Crabb DB, 2020^54^ | ΔT from guidelines recommended time for rhythm check (s) | 12.8 (n.a.) | 17.6 (n.a.) | **0.0358** |
| Crabb DB, 2020^54^ | ΔT from guidelines recommended time for adrenaline (s) | 10.2 (n.a.) | 18.5 (n.a.) | 0.0556 |
| Crabb DB, 2020^54^ | ΔT from guidelines recommended time for defibrillation (s) | 8.9 (n.a.) | 16.7 (n.a.) | 0.1121 |
| Crabb DB, 2020^54^ | ΔT from guidelines for aggregate of all events (s) | 11.5 (n.a.) | 17.7 (n.a.) | **0.0021** |
| Grundgeiger T, 2021^48^ | Deviation from person providing chest compression change algorithm (s) | 29 (28) | 59 (68) | n.a. |
| Grundgeiger T, 2021^48^ | Deviation from heart rhythm algorithm (s) | 12 (11) | 31 (31) | n.a. |
| **Study** | **Secondary Outcomes** | **Intervention** | **Control** | ***p*** |
|  | **CPR quality metrics** |  |  |  |
| Hejjaji V, 2020^55^ | Compression fraction (%)^b^ | 90.9% (2.3%) | 89.0% (5.0%) | **0.007** |
| Grundgeiger T, 2021^48^ | No-flow fraction (%)^b^ | 13.28% (4.09) | 14.06% (4.40%) | n.a. |
| Grundgeiger T, 2021^48^ | Average CC depth (cm) ^b^ | 4.6 (1.0) | 4.0 (1.0) | n.a. |
| Grundgeiger T, 2021^48^ | Average CC rate (min^-1^) ^b^ | 121 (7) | 119 (12) | n.a. |
|  | **Non-technical skills** |  |  |  |
| Lelaidier R, 2017^51^ | Ottawa GRS (7-42)^b^ | 33.7 (4.4) | 30.9 (4.9) | **<0.001** |
| Donzé P, 2019^33^ | Ottawa GRS (0-42)^a,b^ | 36 (33-39) ^a,^, 36.0 (3.96)^b^ | PCA: 32.8 (4.98)^b^  No support: 30 (27-34)^a^, 29.9 (5.95)^b^ | n.s.  **0.004** |
| Shear TD, 2019^53^ | ANTS score^b^ | 42.50 (7.51) | 43.39 (7.69) | 0.6319 |
|  | **Workload** |  |  |  |
| Grundgeiger T, 2021^48^ | NASA-Task Load Index (Raw Task Load Index)^b^ | Physician 8.92 (4.93)  Nurse 8.08 (3.99) | Physician 10.50 (3.83)  Nurse 8.00 (5.25) | 0.277  0.650 |

*Abbreviation: %=percentage, ACLS=advanced cardiac life support, ANTS=Anaesthesia Non-Technical Skills, CA=cardiac arrest, CAST=cardiac arrest simulation tests, CC= chest compression, cm_ centimetres, CPR=cardiopulmonary resuscitation, ERC=European Resuscitation Council, ESC=European Society of Cardiology, GRS=global rating scale, min= minutes, n.a.=not available, n.s.=not significant, PCA=paper cognitive aid, s=seconds, SFAR=French Society of Anaesthesia and Intensive Care. ^a^reported as median (inter-quartile range), ^b^reported as mean (standard deviation), ^c^reported as mean and 95% confidence interval, ^d^reported as median and range, *the same randomized controlled trial with two different cohorts that conducted two different scenarios of cardiac arrest.*
