# Supplementary material for: The impact of cognitive aids on resuscitation performance in in-hospital cardiac arrest scenarios: a systematic review and meta-analysis
Source: Intern Emerg Med. 2022 Aug 29;17(7):2143–58. doi: 10.1007/s11739-022-03041-6 (PMC9420676; doi:10.1007/s11739-022-03041-6)
Supplement: Supplementary file 4 — Supplementary file4 (DOCX 24 KB) [file 11739_2022_3041_MOESM4_ESM.docx]

**The impact of cognitive aids on resuscitation performance in simulated in-hospital cardiac arrest scenarios: a systematic review and meta-analysis**

**Supplementary file 4. Summary of adult studies results on additional primary outcomes**

| **Outcome** | **Study** | **Intervention** | **Control** | **p** |
| --- | --- | --- | --- | --- |
| **Number of deviations** |  |  |  |  |
| Number of errors of commission (not omission)^a^ | Field LC, 2014^36^ | 2.5 (n.a.) | 3.8 (n.a.) | **<0.012** |
| Number of errors ^b^ | Hejjaji V, 2020^55^ | 0.3 (0.6) | 1.0 (1.3) | **<0.001** |
| Number in errors (max 15) | Hall C, 2020^56^ | 27.87% (2.95%)^b^  33.3% (13.3%-40.0%)^c^ | 41.33% (3.82%)^b^  40.0% (13.3-60.0%)^c^ | n.a. |
| **Specific deviations** |  |  |  |  |
| No recognition CA and VF | Shear TD, 2019^53^ | 20% | 28.9% | 0.3975 |
| Incorrect rhythm identification | Hejjaji V, 2020^55^ | 7/53 | 10/53 | n.a. |
| Incorrect number of rhythm checks | Grundgeiger, 2021^48^ | 3.1% | 19.4% | n.a. |
| Incorrect number of chest compressor changes | Grundgeiger, 2021^48^ | 59.4% | 80.6% | n.a. |
| No chest compression | Shear TD, 2019^53^ | 0% | 10.5% | 0.067 |
| Pulse check at irregular intervals | Hejjaji V, 2020^55^ | 0/53 | 7/53 | n.a. |
| Checked blood pression during chest compressions | Hejjaji V, 2020^55^ | 3/53 | 3/53 | n.a. |
| Interruptions during CPR | Shear TD, 2019^53^ | 100% | 94.7% | 0.2177 |
| No adrenaline | Jones I, 2019^52^ | 1/8 | 4/8 | n.a. |
|  | Shear TD, 2019^53^ | 40% | 68.4% | 0.0191 |
| No adrenaline as first drug given | Schneider AJ, 1995^50^ | 0/20 | 2/19 | n.s. |
| Incorrect timing of first dose adrenaline | Hejjaji V, 2020^55^ | 2/53 | 13/53 | n.a. |
| Incorrect timing of second dose of adrenaline | Hejjaji V, 2020^55^ | 7/53 | 20/53 | n.a. |
| Deviation from adrenaline algorithm (3-5 min) | Grundgeiger, 2021^48^ | 59.4% | 74.2% | n.a. |
| Incorrect number of adrenaline administrations | Grundgeiger, 2021^48^ | 18.7% | 12.9% | n.a. |
| Incorrect adrenaline administration and failure to defibrillate | Hejjaji V, 2020^55^ | 3/53 | 10/53 | n.a. |
| No amiodarone | Jones I, 2019^52^ | 0/8 | 1/8 | n.a. |
|  | Hejjaji V, 2020^55^ | 4/53 | 13/53 | n.a. |
|  | Grundgeiger, 2021^48^ | 18.8% | 25.8% | n.a. |
| Failed second dose of amiodarone | Hejjaji V, 2020^55^ | 9/53 | 19/53 | n.a. |
| Incorrect first dose of amiodarone | Hejjaji V, 2020^55^ | 2/53 | 6/53 | n.a. |
| Incorrect second dose of amiodarone | Hejjaji V, 2020^55^ | 4/53 | 1/53 | n.a. |
| Incorrect amiodarone timing | Hejjaji V, 2020^55^ | 5/53 | 5/53 | n.a. |
| No lidocaine as second drug given | Schneider AJ, 1995^50^ | 1/20 | 5/19 | n.s. |
| Incorrect lidocaine dose | Schneider AJ, 1995^50^ | 1/20 | 8/19 | **0.015** |
| Used atropine to treat for PEA | Hejjaji V, 2020^55^ | 2/53 | 5/53 | n.a. |
| No administration of magnesium sulfate | Schneider AJ, 1995^50^ | 8/20 | 17/19 | 0.003 |
| No administration of procainamide | Schneider AJ, 1995^50^ | 14/20 | 16/19 | n.s. |
| Incorrect settings for defibrillation | Schneider AJ, 1995^50^ | 0/20 | 0/19 | n.s. |
| No lowering the head of the OR table | Schneider AJ, 1995^50^ | 20/20 | 19/19 | n.s. |
| No ventilation with oxygen | Schneider AJ, 1995^50^ | 0/20 | 0/19 | n.s. |
| No shock | Jones I, 2019 ^52^ | 0/8 | 1/8 | n.a. |
|  | Shear TD, 2019^53^ | 13.3% | 94.7% | **<0.0001** |
| Unsafe shock | Jones I, 2019^52^ | 3/8 | 0/8 | n.a. |
| Inappropriate defibrillation for PEA | Hejjaji V, 2020^55^ | 1/53 | 7/53 | n.a. |

*Abbreviation: %=percentage, ΔT=difference in time, CA=cardiac arrest, CPR=cardiopulmonary resuscitation, n.a.=not available, n.s.=not significant, OR=operating room, PEA=pulseless electrical activity, s=seconds, VF=ventricular fibrillation. ^a^reported as mean and 95% confidence interval, ^b^reported as mean (standard deviation), ^c^reported as median and range.*
