# Supplementary material for: The impact of cognitive aids on resuscitation performance in in-hospital cardiac arrest scenarios: a systematic review and meta-analysis
Source: Intern Emerg Med. 2022 Aug 29;17(7):2143–58. doi: 10.1007/s11739-022-03041-6 (PMC9420676; doi:10.1007/s11739-022-03041-6)

**The impact of cognitive aids on resuscitation performance in simulated in-hospital cardiac arrest scenarios: a systematic review and meta-analysis**

**Supplementary file 5. Funnel plots**

**a. Electronic cognitive aid versus no cognitive aid**


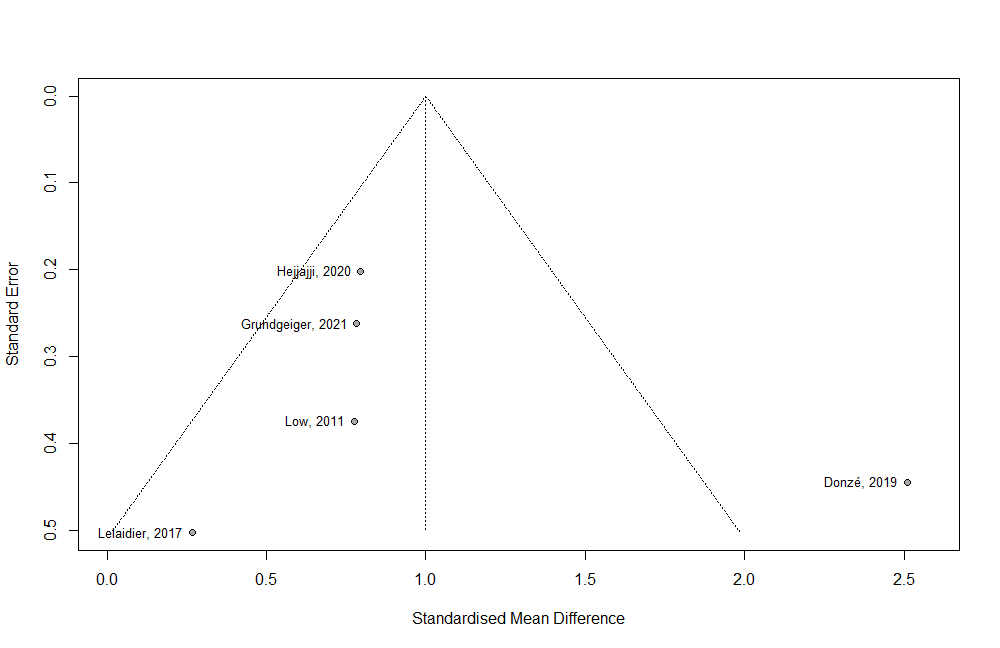


**b. Paper-based cognitive aid versus no cognitive aid**


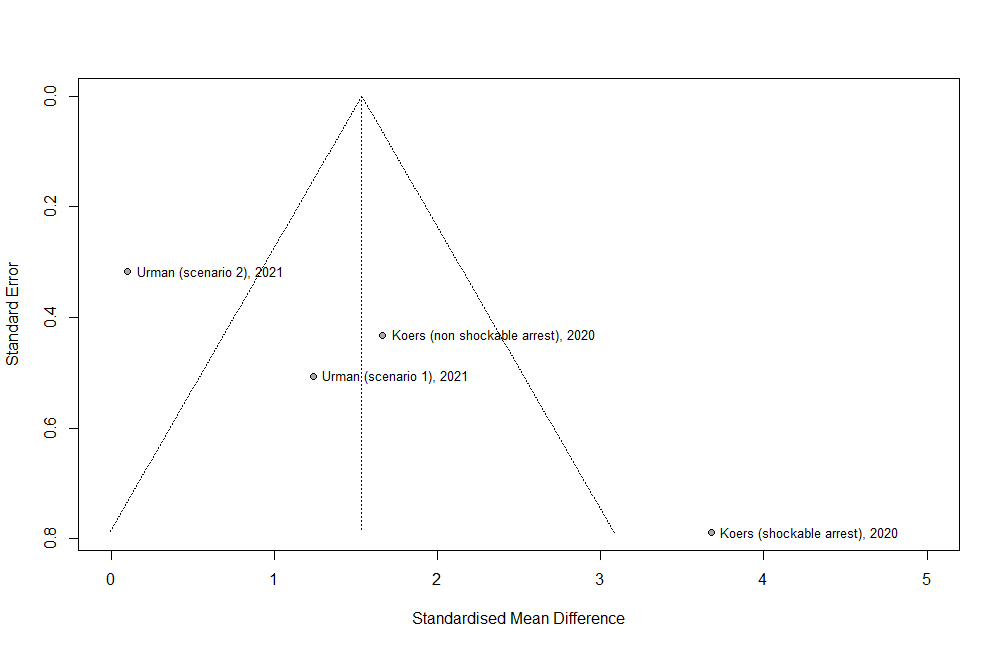


**c. Electronic and paper-based cognitive aid versus no cognitive aid**


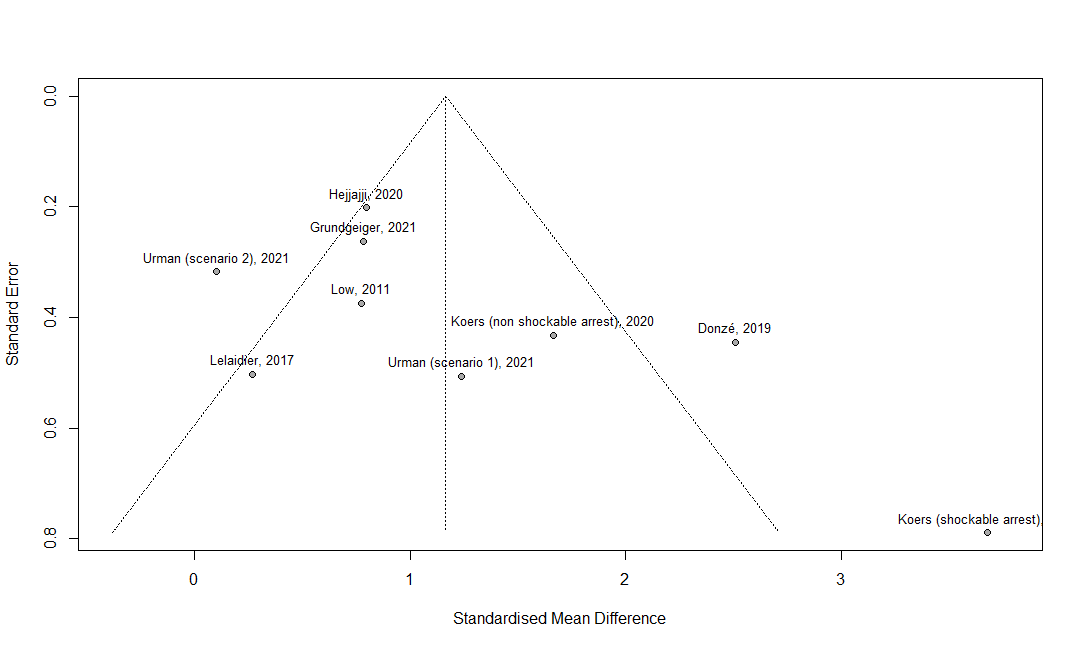


**d. Electronic cognitive aid versus paper-based cognitive aid**


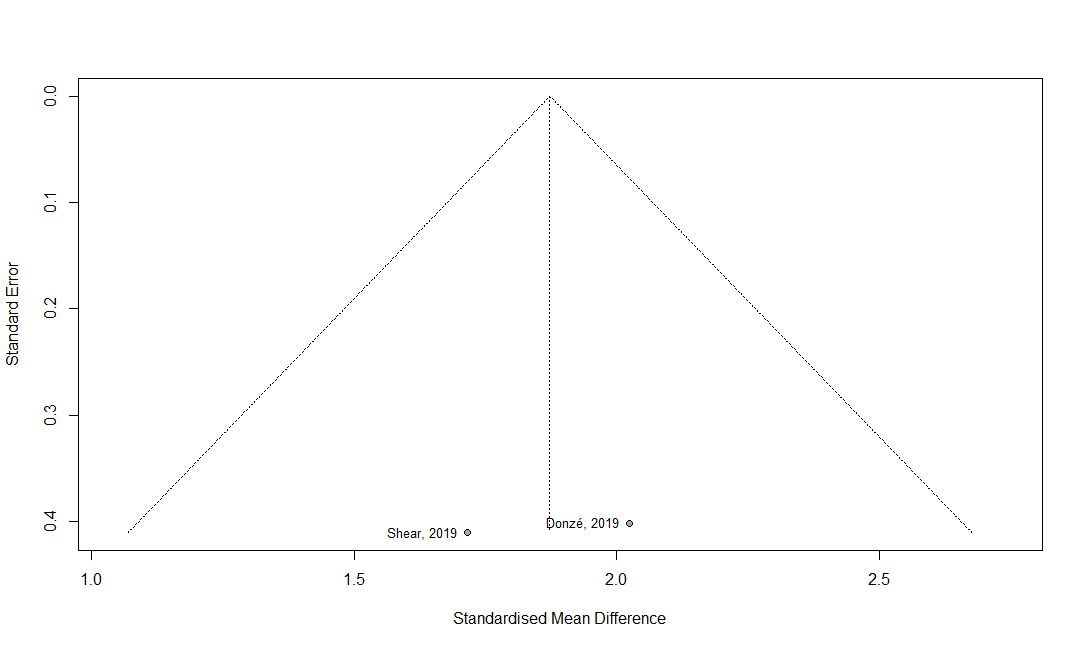

Supplement: Supplementary file 5 — Supplementary file5 (DOCX 116 KB) [file 11739_2022_3041_MOESM5_ESM.docx]
