# Supplementary material for: The impact of cognitive aids on resuscitation performance in in-hospital cardiac arrest scenarios: a systematic review and meta-analysis
Source: Intern Emerg Med. 2022 Aug 29;17(7):2143–58. doi: 10.1007/s11739-022-03041-6 (PMC9420676; doi:10.1007/s11739-022-03041-6)

**The impact of cognitive aids on resuscitation performance in simulated in-hospital cardiac arrest scenarios: a systematic review and meta-analysis**

**Supplementary file 6.** **Meta-analysis of additional outcomes from the paediatric studies**

**a) Time to the second defibrillation**


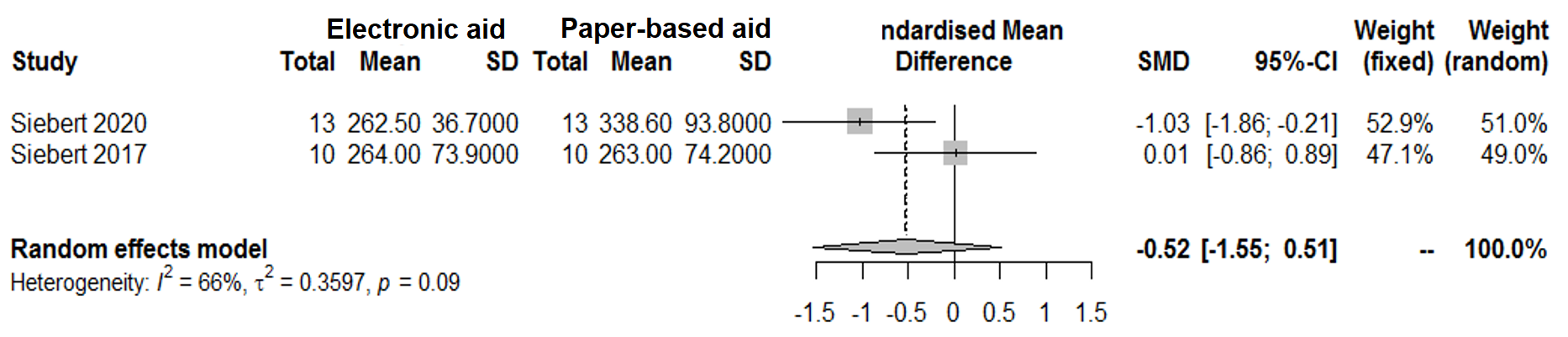


**b) Time to the third defibrillation**


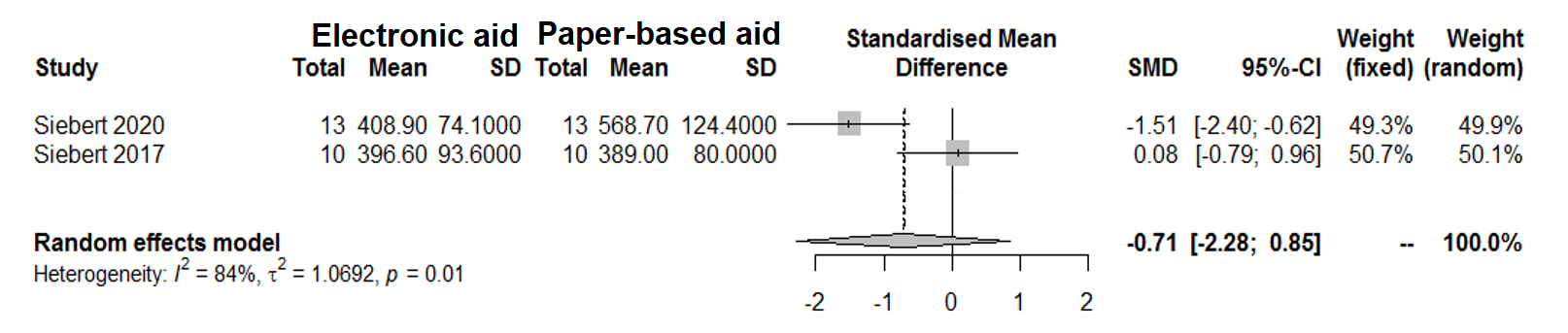


**c) Time to the fourth defibrillation**


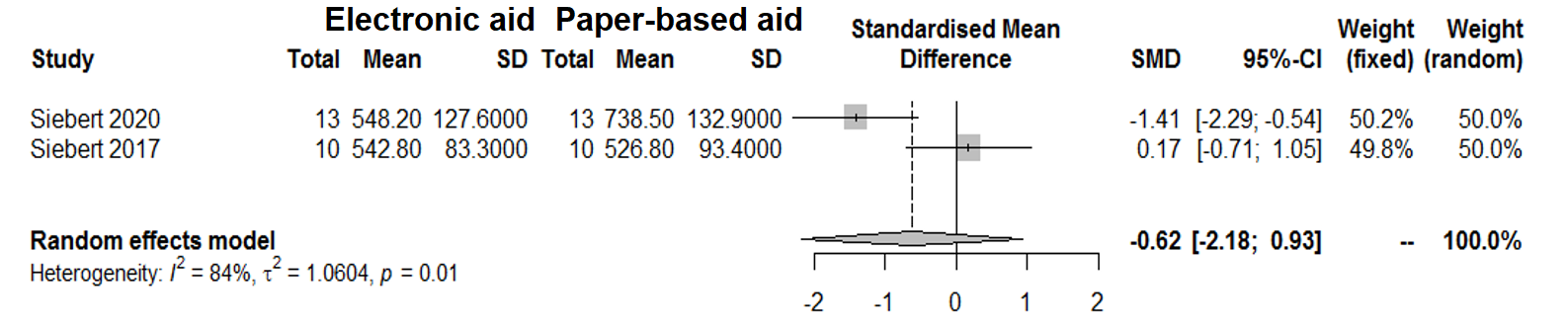


**d) Rate of teams that perform chest compressions within 60 seconds**


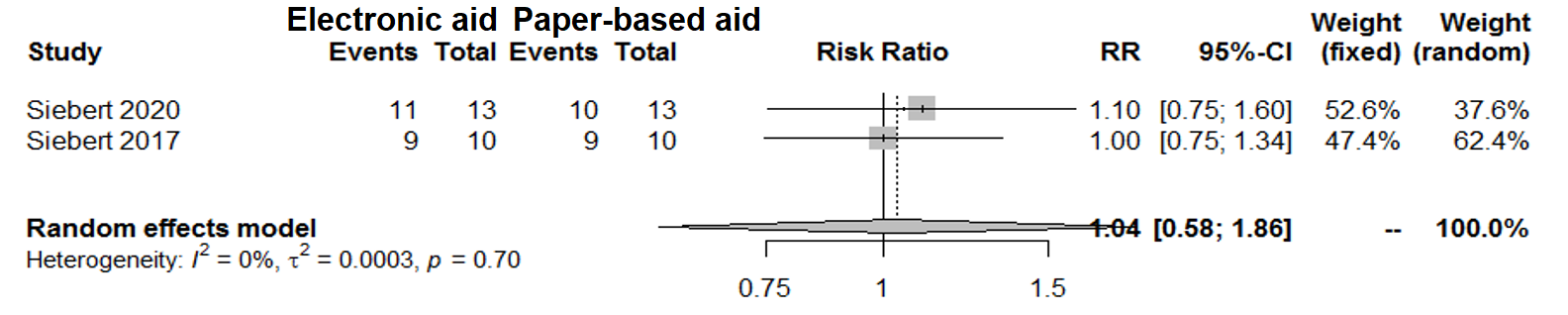


**e) Rate of teams that perform defibrillation within 180 seconds**


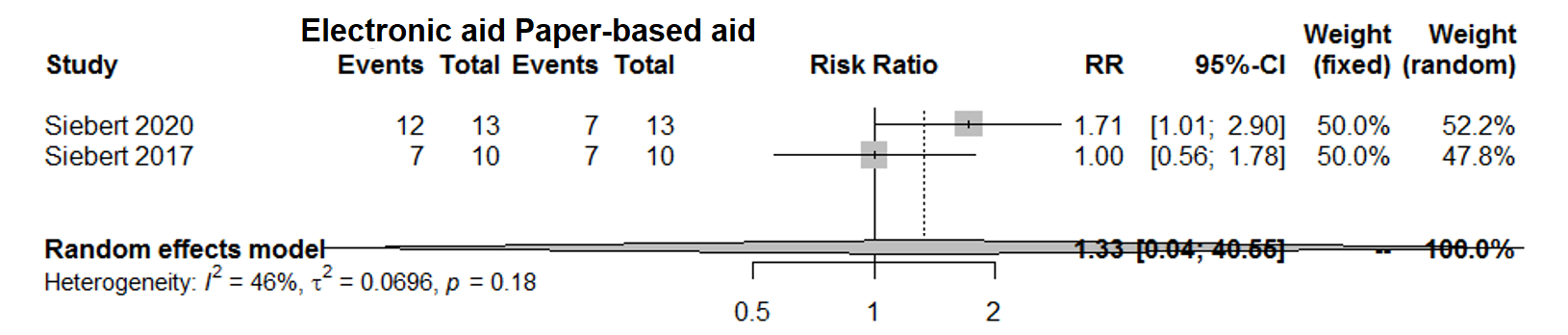


**f) Participants’ perceived stress**


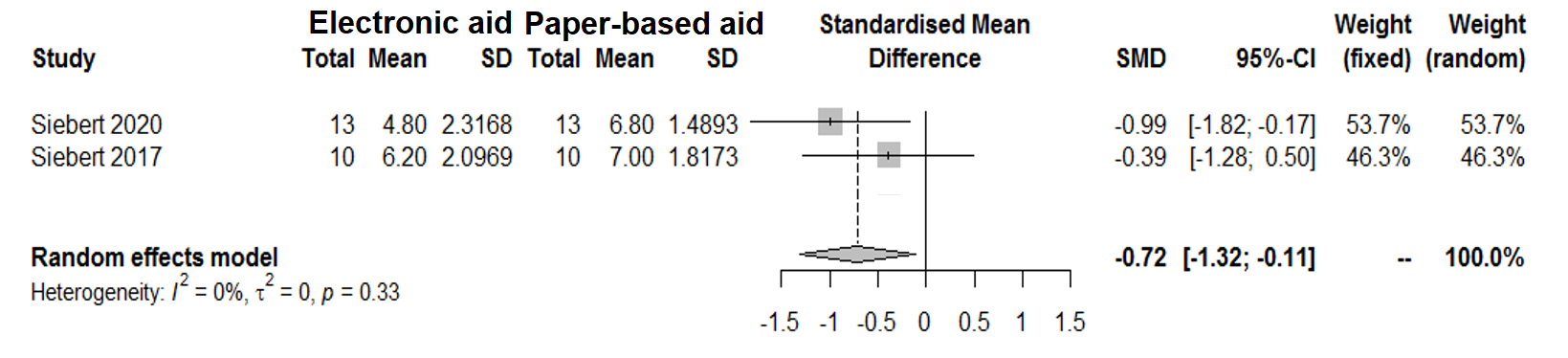

Supplement: Supplementary file 6 — Supplementary file6 (DOCX 3111 KB) [file 11739_2022_3041_MOESM6_ESM.docx]
