# Supplementary material for: The impact of cognitive aids on resuscitation performance in in-hospital cardiac arrest scenarios: a systematic review and meta-analysis
Source: Intern Emerg Med. 2022 Aug 29;17(7):2143–58. doi: 10.1007/s11739-022-03041-6 (PMC9420676; doi:10.1007/s11739-022-03041-6)
Supplement: Supplementary file 7 — Supplementary file7 (DOCX 26 KB) [file 11739_2022_3041_MOESM7_ESM.docx]

**The impact of cognitive aids on resuscitation performance in simulated in-hospital cardiac arrest scenarios: a systematic review and meta-analysis**

**Supplementary file 7. Summary of paediatric studies results**

|  | **Siebert JN, 2017** ^30^ | | | **Siebert JN, 2020** ^31^ | | |
| --- | --- | --- | --- | --- | --- | --- |
| **Outcome** | **Intervention**  **n=10** | **Control**  **n=10** | ***p*** | **Intervention**  **n=13** | **Control**  **n=13** | ***p*** |
| **Adherence to guidelines** | | | | | | |
| % of teams following the correct AHA sequence (%) | 60% | 40% | 0.66 | 92% | 23% | **0.001** |
| Errors in defibrillation doses (%) | 12.5% | 65% | **<0.001** | 2% | 15% | **<0.03** |
| Shock overdoses (>100 J) (%) | 2.5% | 53% | **<0.001** | n.a. | n.a. | - |
| Errors in adrenaline/amiodarone dose concentrations (%) | 0% | 0% | 1 | n.a. | n.a. | - |
| Errors in shock/drug doses (%) | n.a. | n.a. | - | 1% | 14% | **0.005** |
| % of teams that did not recognized initial rhythm (%) | 0% | 0% | n.s. | 2% | 63% | **<0.001** |
| % of teams that delivered the correct number of shocks (%) | 20% | 30% | n.a. | n.a. | n.a. | - |
| % of teams defibrillated within 180 s (%) | 70% | 70% | n.s. | 92% | 54% | n.a. |
| % of teams started CC within 60 s (%) | 90% | 90% | n.s. | 85% | 77% | n.a. |
| % of incorrect shocks (%) | n.a. | n.a. | - | 2% | 2% | n.s. |
| **Time to perform specific resuscitation actions^a^** | | | | | | |
| Initiation of CPR (s) | 28.0 (22.9) | 25.6 (17.8) | 0.80 | 32.1 (22.1) | 48.5 (71.1) | 0.91 |
| 1st shock (s) | 146.2 (43.5) | 145.7(75.1) | 0.99 | 121.4 (26.7) | 211.5 (81.2) | **<0.001** |
| 2nd shock (s) | 264.0 (73.9) | 263.0 (74.2) | 0.98 | 262.5 (36.7) | 338.6 (93.8) | **0.01** |
| 3rd shock (s) | 396.6 (93.6) | 389.0 (80.0) | 0.86 | 408.9 (74.1) | 568.7 (124.4) | **<0.001** |
| 4th shock (s) | 542.8 (83.3) | 526.8 (93.4) | 0.71 | 548.2 (127.6) | 738.5 (132.9) | **<0.001** |
| ΔT initiation of CC - first shock (s) | n.a. | n.a. | - | 89.3 (n.a) | 163.0 (n.a.) | **0.002** |
| Adrenaline administration (s) | 317.3 (62.6) | 295.8 (97.7) | 0.59 | 269.1 (74.8) | 287.2 (82.9) | 0.56 |
| Amiodarone administration (s) | 450.1 (53.6) | 492.7 (106.5) | 0.28 | 455.5 (106.9) | 598.2 (154.7) | **0.01** |
| Intraosseous access (s) | n.a. | n.a. | - | 187.2 (45.4) | 183.0 (71.3) | 0.86 |
| **Workload evaluation^b^** | | | | | | |
| Perceived stress pre-scenario (0-10) | n.a. | n.a. | - | 5.3 (4.0-6.6) | 5.1 (3.9-6.3) | 0.78 |
| Perceived stress post-scenario (0-10) | 6.2 (4.7-7.7) | 7.0 (5.7-8.3) | 0.38 | 4.8 (3.4-6.2) | 6.8 (5.9-7.8) | **0.01** |

*Abbreviations: %=percentage, AHA=American Heart Association, CC=chest compressions, CPR=cardiopulmonary resuscitation, ΔT=difference of time, J=joules, n=number of teams, n.a.=not available, n.s.=not significant, s=seconds. ^a^reported as mean (standard deviation); ^b^reported as mean (95% confidence interval).*
